# Prevalence and characteristics of long COVID-19 in Jordan: A cross sectional survey

**DOI:** 10.1371/journal.pone.0295969

**Published:** 2024-01-26

**Authors:** Marya Obeidat, Abdulmalek Abu Zahra, Farah Alsattari

**Affiliations:** Department of Medical Laboratory Sciences, Faculty of Applied Medical Sciences, Jordan University of Science and Technology, Irbid, Jordan; King Saud University College of Medicine, SAUDI ARABIA

## Abstract

Early in the pandemic, the spread of the emerging virus SARS-CoV-2 was causing mild illness lasting less than two weeks for most people, with a small proportion of people developing serious illness or death. However, as the pandemic progressed, many people reported suffering from symptoms for weeks or months after their initial infection. Persistence of COVID-19 symptoms beyond one month, or what is known as long COVID-19, is recognized as a risk of acute infection. Up to date, information on long COVID-19 among Jordanian patients has not been reported. Therefore, we sought to conduct this cross-sectional study utilizing a self-administered survey. The survey asks a series of questions regarding participant demographics, long COVID-19 symptoms, information about pre-existing medical history, supplements, vaccination history, and symptoms recorded after vaccination. Chi square analysis was conducted on 990 responders, and the results showed a significant correlation (*P*<0.05) between long COVID-19 syndrome and age, obesity, chronic illness, vitamin D intake, number of times infected by COVID-19, number of COVID-19 symptoms and whether the infection was pre or post vaccination. The long-term symptoms most enriched in those with long COVID-19 were tinnitus (73.4%), concentration problems (68.6%) and muscle and joint ache (68.3%).A binomial logistic regression analysis was done to explore the predictors of long COVID-19 and found that age 18–45, marital status, vitamin D, number of COVID-19 symptoms and signs after vaccination are positive predictors of long COVID-19, while zinc intake is a negative predictor. Although further studies on long-term persistence of symptoms are needed, the present study provides a baseline that allows us to understand the frequency and nature of long COVID-19 among Jordanians

## Introduction

COVID-19, a viral respiratory illness caused by SARS-CoV-2, has been one of the major pandemics world-wide resulting in global healthcare crises and strained healthcare resources. COVID-19 has been recognized as a devastating disease with a broad range of manifestations, ranging from asymptomatic infection to an acute respiratory distress syndrome and multi-organ failure [1]. SARS-CoV-2 is known to infect the respiratory tract, but the viral infection and immune response may expand and affect many other organs, which can lead to heart failure, kidney, and liver damage, as well as hyper inflammatory response of the body [2, 3]. Although most COVID-19 patients undergo uncomplicated recoveries, some patients develop long-lasting symptoms even after recovering from the acute illness [4]. The term longCOVID-19 refers to signs and symptoms that continue for more than 4 weeks post-recovery and are not explained by an alternative diagnosis [5]. Therefore, it is important to identify longCOVID-19 symptoms and consequences, which can help us support and manage patients, especially vulnerable patients (e.g., front-line workers and elderly) and patients with underlying conditions (e.g., cancer and cardiovascular diseases).

The development of many post-recovery symptoms on recovered COVID-19 patients has become a serious concern. However, there is little we know about the clinical sequelae that may persist after recovery. Numerous reports have shown that long COVID-19 encompasses a variety of long-term symptoms in multiple systems including respiratory, cardiovascular, gastrointestinal, neurological, and psychiatric systems [6]. A prospective study of 1007 patients conducted 12 weeks from the first COVID-19 diagnosis in Ankara, reported that 87% of recovered patients continued to suffer from a variety of symptoms, with more than half of the patients (51%) having three or more symptoms; fatigue, myalgia, and loss of weight being the most frequent (overall 29.3%) followed by respiratory symptoms (25.4%) [7]. Furthermore, a recent longitudinal cohort of 143 patients observed after recovery from COVID-19 in Italy, reported that 87% had persistence of at least one symptom, with fatigue (53%), dyspnoea (43%), joint pain (27%) and chest pain (22%) being the most common [4].

Many concerns regarding neurological complications of COVID-19 have been increasingly reported, including altered mental status, encephalitis, and primary psychiatric diagnoses [8]. These symptoms arise during the course of infection, but their long-term consequences on COVID-19 patients are not fully understood. Severely affected COVID-19 patients have been observed to have elevated levels of proinflammatory cytokines and acute respiratory dysfunction, which are known to develop cognitive decline [2]. Many studies have been reporting that systemic inflammation promotes neurodegenerative diseases and cognitive decline [9], thus, increasing the probability of recovered COVID-19 patients to experience neurodegeneration in the upcoming years [9].

COVID-19 has a significant impact on people’s lifestyle, causing high levels of psychological distress, anxiety, and mood alterations [10]. The fear of contracting the virus and adapting to the new norms has been perceived as traumatic events [11]. Consequently, they can increase the risk factor of developing mental diseases, which can potentially lead to depression, anxiety, and post-traumatic stress disorder (PTSD) symptoms [12]. It has been reported that frontline workers experience significantly higher levels of mental conditions, such as insomnia and anxiety, compared to second-line workers with depression in 50.4%, anxiety in 44.6%, and insomnia in 34.0% of frontline workers [13]. A recent study in Italy explored the impact of prolonged exposure to COVID-19 distress on mental health status in non-frontline health care workers. This study showed that even dermatologists, although being non-frontline workers, have been widely exposed to psychopathological symptoms, such as depression, anxiety, and PTSD [10]. Resilience has been reported as the most important protective factor from developing any of the symptoms [10]. Therefore, early detection of those at risk of developing severe mental impairment may be significantly important for preventing and reducing the risk of any psychopathological symptoms.

Although vaccination may reduce the chances of getting COVID-19, it is not clear if it protects against longCOVID-19. A recent study showed that fully vaccinated individuals who developed acute infection were 49% less likely to report longCOVID-19 symptoms as compared to unvaccinated individuals [14]. However, COVID-19 vaccination does not provide full protection against long COVID-19 symptoms. COVID-19 vaccines show high efficiency in clinical trials, but some people still become infected with SARS-CoV-2 after vaccination [14]. Vaccines reduce the risk of long COVID-19 by lowering the chances of contracting COVID-19 in the first place, although, for people who experience breakthrough infection vaccination might only halve the risk of acquiring long COVID-19 [14, 15]. Understanding the prevalence of long COVID-19 among vaccinated individuals provides clues about what causes lingering COVID-19 symptoms even after recovering from the acute infection.

Here, we designed a special questionnaire to examine the health status of long COVID-19 patients and the prevalence of their persistent symptoms [4, 16, 17]. To the best of our knowledge, this survey is the first of its kind in Jordan. Analysis of the obtained data provided an impression of the long-term complications associated with COVID-19 infection and identified the most common ones among the Jordanian population as well as highlighted the main factors that could be associated with such complications. Thus, this study may provide a useful insight to develop better strategies to reduce and manage long COVID-19 complications.

## Materials and methods

### Study design

This cross-sectional study was conducted in Jordan during the period (April 16-August 17, 2022). It utilized a self-administered questionnaire that was written in Arabic and English languages and was constructed using Google forms (S1 File). The questionnaire was designed to address long COVID-19 status and factors that may associate with it among Jordanians. It included questions regarding COVID-19 symptoms, pre-existing medical history, treatment and supplements, COVID-19 vaccination history, and symptoms recorded after vaccination. We adopted the definition of long COVID-19 that refers to individuals experiencing at least one symptom longer than four weeks [5]. The study included Jordanian adults who were previously infected with COVID-19. The questionnaire was first piloted and revised based on the comments.

### Study sample

The study sample was recruited through electronic distribution of the survey via WhatsApp and Facebook platforms. Facebook was the primary social media platform for outreach as it is the most common social media platform used in Jordan (https://gs.statcounter.com/social-media-stats/all/jordan), last access on October 12, 2023. Specifically, we leveraged Facebook groups that were purposefully created to represent and engage with distinct neighbourhoods within Jordan. In addition, we complemented our efforts by collecting responses in the field, focusing on identified hotspots where we anticipated higher foot traffic and engagement. Approximately 10–15% of our survey data was gathered through face-to-face interactions, allowing us to capture valuable insights from individuals who may not have been easily accessible through online means. This hybrid approach aimed to maximize the representativeness of our sample and enhance the reliability of our research findings. Furthermore, to ensure a comprehensive and inclusive approach, we enlisted the assistance of individuals residing in various Governorates across the Kingdom of Jordan.

The required sample size was determined using an online sample size calculator (calculator.net). The confidence interval, margin of error, and response distribution were 95%, 3%, and 50%, respectively. The target population size was 1,730,167, which is the total number of confirmed COVID-19 cases in Jordan by August 17, 2022 (https://www.worldometers.info), last access October 12, 2023. The recommended sample size was 1067; however, we were able to receive data from only 1015 responders. Twenty-five responders who were 12–17 years of age and one who did not complete the survey were excluded from the analysis.

### Ethics

The study was approved by the institutional review board (IRB) committee at Jordan university of science and technology and King Abdullah university hospital (Ref #: 46/148/2022). An informed consent pagethat preceded the start of the survey clarified the main goal of the study. Participants who submitted the responses have read and approved the written consent. Participation was completely voluntary and restricted to those who were previously infected with COVID-19and the questionnaire data were anonymized to ensure privacy and confidentiality. In addition, participants did not have to answer any questions they did not want to; options indicated that the absence of a response for any given question is intentional, or participantscan choose not to answer them.

### Statistical analysis

Data were analyzed using SPSS version 22 (IBM Inc., Armonk, New York, United States). Percentages were used to describe categorical data. Chi-square analysis was employed to test the correlation between independent variables and long COVID-19, and binomial logistic regression was utilized to predict the relationship between independent variables and long COVID-19. *P*< 0.05 was considered significant.

## Results

### Participants’ demographics

Table 1 demonstrates the sample’s demographics of 990 participants, 71.0% were females, while 29.0% were males. Most participants fell under the age bracket of 18–34 (45.1%), followed by 35–44 (24.3%), 45–54 (16.9%), and 55 or more (13.7%). In terms of marital status, the majority were single (61.1%), followed by married (35.5%) and others that include divorced or widowed (3.4%). More than half of the participants were non-employed (56.5%), while 43.5% were employed either in the private sector (23.2%) or the government sector (20.3%). Regarding smoking habits, 28.4% of the participants were smokers, while 71.6% were non-smokers. Most of the participants were non-obese (81.8%) and received the first and second doses of COVID-19 vaccines (95.4% and 92.8%, respectively), while 16.3% received the third dose. Only 27.4% fell under the category of long COVID-19, while 72.6% did not report long COVID-19 symptoms.

**Table 1:** Demographics of the study participants. (n = 990).

Category	Count	Percentage
Male	287	29.0%
Female	703	71.0%
**Age groups **		
18–34** **	446	45.1%
35–44** **	241	24.3%
45–54** **	167	16.9%
55 or More	136	13.7%
**Marital status **		
Single** **	605	61.1%
Married** **	351	35.5%
Other** **	34	3.4%
**Employment**		
Non-employed** **	559	56.5%
Employed in government sector	201	20.3%
Employed in private sector	230	23.2%
**Smoking**		
Yes	709	71.6%
No** **	281	28.4%
**Obesity**		
No** **	810	81.8%
Yes	180	18.2%
**COVID-19 vaccine history **		
First dose** **	944	95.4%
Second dose** **	919	92.8%
Third dose** **	161	16.3%
**Long COVID-19 (> 1 month) **		
No** **	719	72.6%
Yes** **	271	27.4%

n: number of participants

### The correlation between participants’ characteristics and long COVID-19

Table 2 shows the results of chi-square analyses that examine the relationship between the demographic variables and long COVID-19. Only age and obesity significantly correlated with long COVID-19.The findings indicated that obese participants were less likely to develop long COVID-19 (n = 61, 22.5%) than non-obese (n = 210, 77.5%), (*P* = .034), and that the proportion of participants who had long COVID-19 decreased with age (*P* = 0.05).

**Table 2:** The association between participants’ demographics and long COVID-19. (n = 990).

Category	No long COVID-19 n (%)	Long COVID-19 n (%)	Chi-Square Value	*P*-value
**Sex **			0.147	0.695
Male** **	206(71.8)	81(28.2)		
Female** **	513(73.0)	190(27.0)		
**Age (years) **			7.795	**0.05 **
18–34** **	322(72.2)	124(27.8)		
35–44** **	190(78.8)	51(21.2)		
45–54** **	116(69.5)	51(21.2)		
55 or more** **	91(66.9)	45(33.1)		
**Marital status**			3.377	0.185
Single	442(73.1)	163(26.9)		
Married** **	257(73.2)	94(26.8)		
Other	20(58.8)	14(41.2)		
**Employment **			3.250	0.197
Non employed** **	418(74.8)	141(25.2)		
Private sector	139(68.7)	63(31.3)		
Government sector	163(70.9)	67(29.1)		
**Smoking **			0.029	0.874
Non-Smoker** **	516(72.2)	193(27.2)		
Smoker** **	203(72.2)	78(27.8)		
**Obesity **			4.697	**0.034 **
No** **	600(74.0)	210(26.0)		
Yes** **	119(66.1)	61(26.0)		

n: number of participants

### COVID-19 symptoms distribution among the study participants

**Fig 1:**
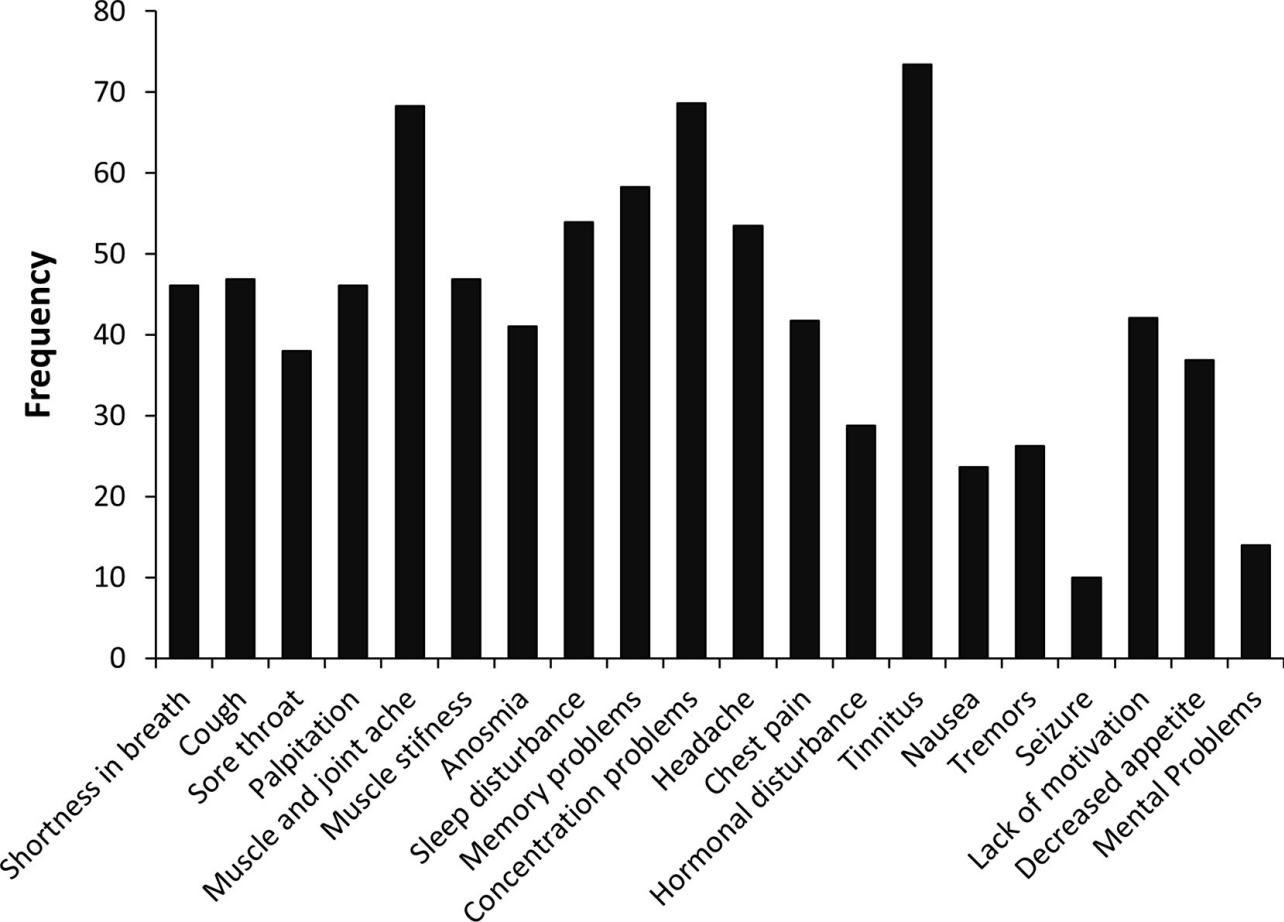
The frequency of long COVID-19 symptoms.

To further characterize the status of long COVID-19 in our study, we asked the participants questions regarding a set of 19 symptoms associated with COVID-19 infection. Fig 1 demonstrates the frequency of each symptom. As shown, the most prevalent symptoms were tinnitus (73.4%), concentration problems (68.6%), and muscle and joint ache (68.3%).

### The correlation between participants’ medical history and long COVID-19

Table 3 represents the results of chi-square analysis investigating the relationship between the participants’ medical history and long COVID-19 status. The medical history included taking the common supplements; vitamin C, B12, D, and zinc, pre-existing chronic illnesses such as pulmonary, cardiovascular, metabolic, and neurological disorders, which were categorized into three groups: none, 1–2 illnesses, and more than 2, number of times participants had COVID-19, hospitalization due to COVID-19 infection, and the number of symptoms participants experienced during COVID-19 infection. The analysis showed that around half of the participants had taken vitamin C (n = 464, 46.9%), while less than a half had vitamin D, B12, or zinc supplements. However, there was a significant association between long COVID-19 and vitamin D intake (*P*<0.001); the number of participants who had taken vitamin D was significantly less in the long COVID-19 group (n = 78, 20.5), as compared to those in the no long COVID-19 group (n = 302, 79.5). Around half of the long COVID-19 participants had 1–2 pre-existing chronic illnesses (n = 132, 48.7%) and only 6.6% (n = 18) had more than 2 chronic diseases. Chi-square analysis revealed that the number of participants who had pre-existing chronic illnesses was significantly higher among the long COVID-19 group, while the ones who did not have any chronic illnesses were found more frequently among the no long COVID-19 group (*P* = 0.006).

**Table 3:** The association between medical history and long COVID-19. (n = 990).

Category	No long COVID-19	Long COVID-19	Chi-Square Value	*P*-value
**Vitamin C **			0.185	0.667
No** **	379(72.1)	147(27.9)		
Yes** **	340(73.3)	124(26.7)		
**Zinc **			1.205	0.272
No** **	501(73.7)	179(26.3)		
Yes** **	218(70.3)	92(29.7)		
**Vitamin B12 **			1.568	0.211
No** **	507(71.5)	202(28.5)		
Yes** **	212(75.4)	69(24.6)		
**Vitamin D **			14.545	**<0.001 **
No** **	417(68.4)	193(31.6)		
Yes** **	302(79.5)	78(20.5)		
**Chronic illness **			10.114	**0.006 **
None** **	399(76.7)	121(23.2)		
1–2** **	272(67.3)	132(32.7)		
>2	48(72.7)	18(27.3)		
**Number of COVID-19 infections **			8.617	**0.013 **
1** **	447(75.6)	154(24.4)		
2** **	198(68.5)	91(31.5)		
3** **	44(62.9)	26(37.1)		
**Hospitalization **			1.626	0.235
No** **	679(73.1)	250(26.9)		
Yes** **	40(65.6)	21(34.4)		
**Number of Symptoms**			33.188	**<0.001 **
1–3** **	265(83.1)	54(16.9)		
4–6** **	111(76.6)	34(23.4)		
More than 6** **	343(65.2)	183(34.8)		

n: number of participants

More than half of the participants have been infected by COVID-19 at least once (n = 601, 62.6%), and most of them were not hospitalized (n = 929, 93.8%). At the same time, more than half of them had more than three symptoms during their infection period (n = 671, 67.8%). The analysis revealed no significant association between hospitalization and long-term COVID-19. However, participants who were infected once or had more than 6 symptoms were more likely to have long-term COVID-19 (*P* = 0.013 and *P*<0.001, respectively).

### The correlation between COVID-19 vaccination and long COVID-19

We examined the relationship between COVID-19 vaccine doses and whether the infection was before or after vaccination and long COVID-19 status (Table 4). Most participants had taken one or two COVID-19 vaccine doses (n = 944, 95.4% and n = 919, 92.8%, respectively). While a small percentage had a third dose (n = 161, 16.3%). More than half of them had been infected by COVID-19 before vaccination (n = 533, 53.8%). There was no significant correlation between long COVID-19 and vaccine doses (*P*>0.05), while long COVID-19 was more likely to occur if the infection was before vaccination (n = 164, 60.5%) than post-vaccination (n = 107, 39.0%) (*P* = 0.010). In addition, signs after vaccination were significantly associated with long-term COVID-19 (n = 200, 74.1%), (*P*<0.001).

**Table 4:** The correlation between vaccination history and long COVID-19.

Category	No long COVID-19 n (%)	Long COVID-19 n (%)	Chi-Square Value	*P*-value
**COVID-19 vaccine dose 1 **			0.227	0.615
No** **	32(69.6)	14(30.4)		
Yes** **	687(72.8)	257(27.2)		
**COVID-19 vaccine dose 2 **			0.024	0.890
No** **	51(71.8)	20(28.2)		
Yes** **	668(72.7)	251(27.3)		
**COVID-19 vaccine dose 3 **			1.866	0.178
No** **	595(71.8)	234(28.2)		
Yes** **	124(77.0)	37(23.0)		
**Time of infection **			6.696	**0.010 **
Pre-Vaccination** **	369(69.2)	164(30.8)		
Post-Vaccination** **	350(76.6)	107(23.4)		
**Signs after vaccination **			14.839	<**0.001**
No** **	281(80.1)	70(19.9)		
Yes** **	438(68.7)	200(31.3)		

n: number of participants

### Study predictors of long COVID-19

A binomial logistic regression was performed to determine whether sex, age, marital status, smoking status, employment, vitamins (D, B12, C, zinc) intake, hospitalization, number of COVID-19 symptoms, number of chronic diseases, number of COVID-19 vaccine doses, number of COVID-19 infection, and signs after vaccination were predictors of long COVD-19 in our study population. The regression model explained 10.9% (Nagelkerke R2) of the variance in long COVID-19. The model was statistically significant χ2 (17) = 77.209, p < 0.001, and demonstrated good fitting as Hosmer and Lemeshow goodness of fit test was χ2 (8) = 4.451, p = 0.814.

The odds ratios for long COVID-19 status based on the participant characteristics are illustrated in Table 5. Younger age (18–34 and 35–44) was associated with an increase in the likelihood of having long COVID-19 symptoms by 1.19 and 2.34 units, respectively, when holding all other variables in the model constant. Married participants were more likely to have long COVID-19 (OR = 1.565; 95% CI:1.02–2.41) than single participants. Increased number of COVID-19 symptoms was associated with an increased likelihood of exhibiting long COVID-19. Participants who were on zinc supplements had 0.62 times lower odds of having long COVID-19 (95% CI: 0.42–0.91), while participants who were on vitamin D had 2.17 times higher odds of having long COVID-19 (95% CI: 1.52–3.11). Participants who had signs after vaccination were more likely to have long COVID-19 (OR = 1.688; 95% CI:1.21–2.36).

**Table 5:** Binomial logistic regression analysis.

Variables	B	S.E.	Sig.	Exp(B)	95% C.I. for EXP(B)	
					Lower	Upper
**Sex (female) **	.133	.181	.460	1.143	.802	1.628
**Age (18–34) **	.583	.293	**.047 **	1.792	1.008	3.185
**Age (35–44) **	.850	.275	**.002 **	2.339	1.364	4.012
**Age (45–54) **	.198	.262	.449	1.220	.730	2.038
**Marital status (married)**	.448	.219	**.041 **	1.565	1.018	2.406
**Employment (Non employed) **	-.280	.179	.118	.756	.532	1.074
**Smoking (Smoker) **	.036	.178	.839	1.037	.731	1.470
**Hospitalization**	-.119	.301	.693	.888	.492	1.602
**Number of COVID-19 infraction**	.175	.117	.136	1.191	.946	1.499
**Number of COVID-19 symptoms**	.052	.013	**.000 **	1.054	1.026	1.082
**Number of chronic diseases**	.007	.037	.858	1.007	.936	1.083
**Vitamin C intake (yes) **	.095	.180	.596	1.100	.773	1.565
** Zinc intake (yes) **	-.476	.194	**.014 **	.621	.424	.909
**Vitamin B12 intake (yes) **	.031	.191	.873	1.031	.709	1.498
**Vitamin D intake (yes) **	.775	.183	**.000 **	2.171	1.516	3.110
**Number of Vaccine doses**	-.217	.127	.088	.805	.628	1.033
**Signs after vaccination (yes) **	.524	.171	**.002 **	1.688	1.206	2.362
**Constant **	3.061	.781	.000	.047		

## Discussion

In this cross-sectional study of a cohort of 990 adults surveyed between May 2022 and August 2022 across Jordan, only 27.4% of the participants reported long-term COVID-19 symptoms that persisted for more than one month. We found that obesity and age were significantly associated with long COVID-19. Tinnitus, concentration problems, and muscle and joint ache were the most long COVID-19 associated symptoms. From the reported medical history of long COVID-19 patients, taking vitamin D supplements and having 1–2 chronic illnesses were strongly associated with long COVID-19 symptoms. Our study found that when patients had higher number of COVID-19 infections and symptoms, the risks of developing long COVID-19 increased. Regarding vaccination, the number of vaccine doses taken by patients did not affect the prevalence of long COVID-19, but whether the infection was conducted pre- or post-vaccination had a significant link with long COVID-19 complications. Considering all factors tested in this study, age, marital status, the number of COVID-19 symptoms, zinc and vitamin D intake, and experiencing signs after vaccinationwere the most predictive factors of long COVID-19.

In the present study, the proportion of participants who reported long COVID-19 was relatively less than in previous studies, which was at least 50% [18, 19]. The majority of the sample consisted of females (71.0%) and young adults with ages ranging from 18–34 (45.1%).As shown previous studies, females are more likely to participate in surveys than males [20] and younger people are more likely to participate than older people [21]. However, we observed no significant difference in the frequency of developing long COVID-19 symptoms between the two sexes. This is contradictory with a previous study that included 21,359 participants, where there was a discernible correlation between females and an increased frequency of long COVID-19 symptoms [22]. In addition, many researchers reported that sex may influence the risk and outcomes of COVID-19 infections [23, 24] and suggested female sex as a risk factor for long COVID-19 [19, 25]. Nevertheless, female sex was not a predictor of long COVID-19 in the current study, despite its domination in our sample. The relationship between age and long-COVID-19 symptoms yielded inconclusive results across various studies. For example, two studies demonstrated a positive association between age and long COVID-19 indicating that older age is linked to a higher likelihood of experiencing long COVID-19 symptoms [26, 27]. Conversely, two other studies reported a negative association, suggesting that younger individuals might be more susceptible to such symptoms [19, 28]. Lastly, another study found no significant association between age and long COVID-19 symptoms [29]. These diverse findings emphasize the complexity of the relationship between age and the manifestation of long COVID-19 symptoms. With regards to age, our study found that the age range 18–34 had the highest number of individuals who experienced long COVID-19 symptoms while the age of 55 and older had the lowest number of individuals experiencing long COVID-19 symptoms, and age ranges 18–34 and 35–44 were positive predictors of long COVID-19 status. Our findings are contradictory to some existing literature while in line with others, confirming the remarkable heterogeneous relationship between age and long COVID-19. Understanding the relationship between age and long COVID-19 allows us to provide different age groups with specific protective measures.

The relationship we observed regarding age and long COVID-19 might be explained by several different factors. Only a small number of individuals that participated in the survey were of age 55 years or older. Moreover, there is a higher tendency for young adults to participate in social events and interactions, which increase their exposure to the virus, hence, a higher probability of developing long COVID-19 symptoms. Furthermore, the intense media coverage of the pandemic caused elderly in Jordan to be more cautious and attentive to their overall wellbeing leading to a less severe infection, thus a lower probability of progressing to long COVID-19. Further supporting our results, a study conducted in the US found that older people were as likely to leave their homes as younger people, but people over the age of 50 had less than half the predicted number of close contacts than those who were younger than 30 [30], therefore reducing the likelihood of developing long COVID-19 symptoms.

Smoking, obesity, and socioeconomic deprivation are among previously reported long COVID-19 associated factors [19]. In our cohort, while smoking and employment did not have an impact on long COVID-19 status, obesity was negatively associated with it. This may in part be due to subjective reporting, as our study did not involve a measurement of body mass index (BMI) and depended on the participants’ judgment. Moreover, marital status was not associated with long COVID-19, but being married served as a positive risk factor for it, which is contradictory to a previous questionnaire survey that investigated the relationship of marital status and long COVID-19 in 749 cases and concluded that depressive and memory impairment symptoms were reported more frequently among the unmarried group [31]. This discrepancy may be due to the differences in analysis; we correlated marital status with the persistence of at least one symptom for more than a month, while the former study analyzed the frequency difference for each symptom between the married and unmarried group, hence our finding is more generalized

Several long COVID-19associated symptoms have been identified to affect many body organs and physiological functions and may last for years [32]. In our cohort, we reported the prevalence of 19 symptoms, among which tinnitus, concentration problems, and muscle and joint ache were the most prevalent. According to a Meta analysis that included 2,100 studies, the most prevalent long COVID-19 symptoms were chest abnormalities, concentration difficulty, generalized anxiety, general functional impairment, and fatigue or muscle weakness [18].

The former supplements that were addressed in our survey were chosen based on the recommendations by the national institute of health regarding their potential roles in boosting immunity and ameliorating COVID-19 infection. No significant correlation was found with any of vitamin B12, vitamin C and zinc supplements. These findings are consistent with a meta-analysis that identified 26 studies, where nine studies of 1488 individuals that evaluated vitamin C intake found that it had no significant effect on mortality; intubation rate and length of hospital stay [33]. Another five studies that included 738 individuals showed that zinc supplementation was not significantly associated with mortality [33]. In our study, only vitamin D supplementation showed a significant correlation with long COVID-19 symptoms (P < 0.001). However, when all study variables have been considered, vitamin D and zinc intake positively and negatively impacted long COVID-19 status, respectively. Vitamin D deficiency has been linked to infectious diseases, some types of cancers and chronic inflammation [34]. Additionally, it is related to the severity and mortality of COVID-19 cases, where high prevalence of vitamin D deficiency has been found in patients with COVID-19 with acute respiratory failure [35]. In addition, research that included 55 patients reported that vitamin D deficiency prolonged the recovery from long COVID-19, while zinc deficiency correlated with persistent inflammation [36].

A study published in *BMJ Medicine* found that vaccination against COVID-19 reduces both severity and duration of long COVID-19 [37]. In the present study we asked about the vaccination status against COVID-19 to evaluate whether it correlates with developing long COVID-19 symptoms. There was no significant correlation between vaccine doses (P>0.05) and long COVID-19, which is consistent with a study that was published recently in *Nature* [38]. However, we found significant negative association when COVID-19 infection occurred post-vaccination, suggesting that vaccination reduced the rate of long COVID-19 [39]. A systematic review revealed that six out of eight studies of two or more doses of vaccine given before infection with the SARS-CoV-2 virus found significant reductions in the rates of long COVID-19 [40]. Moreover, a meta-analysis that included 6 studies involving 8,199 long COVID-19 patients revealed that a two-dose vaccination was linked with a lower probability of developing long COVID-19 compared to zero or one-dose vaccination [41]. Nonetheless, individuals suffering from ongoing long COVID-19 symptoms did not report any changes in their symptoms after vaccination [41]. We conclude that most studies suggest that COVID-19 vaccines might have protective effects on long COVID-19, but more robust comparative studies are needed to determine the factors that impact the effectiveness of vaccines in preventing it.

During the recent COVID-19 pandemic, patients with chronic illnesses were exposed to a serious threat. The pandemic created significant barriers to diagnosis and treatment of chronic diseases and limited access to health care facilities. Here, we found that the presence of 1–2 chronic illnesses in our participants positively increased the likelihood of developing long COVID-19. A meta-analysis showed that COVID-19 patients with chronic diseases such as hypertension, diabetes and coronary heart diseases are more likely to progress to severe conditions [42]. Thus, greater attention is needed to discover solutions for better chronic disease management of long COVID-19 patients. Furthermore, we revealed that participants who had more than 6 symptoms or had been infected once withSARS-CoV-2 were more likely to develop long COVID-19, which is not unexpected as the second exposure to the virus is usually milder than the first infection due to the acquired immunity.

## Conclusion

Our study opens doors to analyze the risk factors for progression to long-term complications in COVID-19 patients within the Jordanian population. In aggregate, the results of this cross-sectional study provide valuable information regarding the association of age, obesity, vitamin D intake, the number of COVID-19 infections and symptoms, and the presence of 1–2 chronic illnesses with the likelihood of developing long COVID-19. The findings also support the potential association between the time of infection in relation to vaccination in reducing long COVID-19risk.Themost common clinical symptoms in our study are Tinnitus, concentration problems, and muscle and joint ache. Our results may facilitate early intervention to minimize the effect of long COVID-19 and could contribute to preventing this syndrome within the Jordanian communities. Further investigations on how different variants of the virus cause different long-term sequalae may help us better understand the mechanisms underlying the development of long COVID-19 syndrome.

However, some limitations must be considered when interpreting our current findings. Our study is cross sectional and showed some level of heterogeneity. However, a certain level of heterogeneity across different studies is inevitable, given the large variation that can be present in the settings and patients characteristics. Here, more than half of the participants were females and in the age group 18–45, which may be less representative of the target population. Moreover, we relied on self-report of symptoms rather than physiological or cognitive measures, which might have introduced bias in our results because it relies on participant recall of symptoms, therefore the level of accuracy in filling the survey is not guaranteed. As well as, response to online surveys is affected by access to internet and social platforms, literacy rate, and knowledge in information technology, which might have limited the number of participants who are older than 55 and influenced our findings in regard to age.

## Supporting information

S1 File Long Covid-19 survey. (DOCX)

S2 File Inclusivity-in-global-research-questionnaire. (DOCX)
