# Supplementary material for: Prevalence and characteristics of long COVID-19 in Jordan: A cross sectional survey
Source: PLoS One. 2024 Jan 26;19(1):e0295969. doi: 10.1371/journal.pone.0295969 (PMC10817197; doi:10.1371/journal.pone.0295969)
Supplement: S1 File — (DOCX) [file pone.0295969.s001.docx]

**Prevalence and characteristics of long COVID-19 in Jordan: A cross sectional survey**

We are a research team from Jordan University of science and technology conducting a study to address the long-term effects of COVID-19 among Jordanian patients. To enroll in this study, you should have been infected with COVID-19. We ask you please to answer the survey as precisely as possible. You should know that your answers will only be used for research purpose and that your participation in the study is completely voluntary and that any information you provide will be kept private and handled with extreme confidentiality. You will not be asked to provide your name or any identifying information. We thank you very much and appreciate your participation in this study.

**التاثيرات الصحية طويلة الامد لمرض فيروس كورونا المستجد في الاردن: مسح مقطعي**

ﻧﺣن ﻓرﯾق ﺑﺣﺛﻲ ﻣن ﺟﺎﻣﻌﺔ اﻟﻌﻠوم واﻟﺗﻛﻧوﻟوﺟﯾﺎ اﻻردﻧﯾﺔ ﻧﺟري دراﺳﺔ ﻋﻠﻰ اﻟﺗﺄﺛﯾرات اﻟﺻﺣﯾﺔ طوﯾﻠﺔ اﻻﻣد ﻟﻣرض ﻓﯾروس ﻛوروﻧﺎ 19اﻟﻣﺳﺗﺟد.ﻟﻠﻣﺷﺎرﻛﺔ ﻓﻲ ھذه اﻟدراﺳﺔ ﯾﺟب ان ﺗﻛون ﻗد اﺻﺑت ﺳﺎﺑﻘﺎ ﺑﺎﻟﻛوروﻧﺎ. ﻧرﺟواﻻﺟﺎﺑﺔ ﻋن أﺳﺋﻠﺔ اﻟدراﺳﺔ ﺑﻛل دﻗﺔ،ﻋﻠﻣﺎ ﺑﺎن اﻟﻣﻌﻠوﻣﺎت اﻟﺗﻲ ﺳوف ﺗدﻟﻲ ﺑﮭﺎ ﻟن ﺗﺳﺗﺧدم إﻻ ﻷﻏراض اﻟﺑﺣث اﻟﻌﻠﻣﻲ ﻓﻘط وان اﺷﺗراﻛك ﻓﻲھذه اﻟدراﺳﺔ ھو طوﻋﻲ وﺳوف ﺗﻌﺎﻣل اﻟﻣﻌﻠوﻣﺎت ﺑﻛل ﺳرﯾﺔ وﺧﺻوﺻﯾﺔ وﻟن ﯾطﻠب ﻣﻧك اﻻﻓﺻﺎحﻋن اﺳﻣك اوايﻣﻌﻠوﻣﺎت ﺷﺧﺻﯾﺔ.وﺷﻛرا ﺟزﯾﻼ ﻟﻼﺷﺗراك ﻓﻲھذه اﻟدراﺳﺔ

*Indicates required question

* يشير الى سؤال يجب الاجابة عليه

*اﻟﺟﻧس1. Sex.

*Mark only one oval. اختار اجابة واحدة فقط*


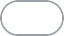
Male ذﻛر

أﻧﺛﻰFemale
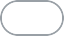


*اﻟﻌﻣر2. Age.

*Mark only one oval. اختار اجابة واحدة فقط*


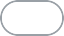
12-17


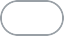
18-24


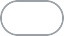
25-34


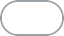
35-44


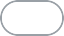
45-54


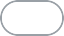
55 and older

*اﻟﺣﺎﻟﺔ اﻻﺟﺗﻣﺎﻋﯾﺔ 3. Marital status.

*Mark only one oval. اختار اجابة واحدة فقط*

أﻋزبSingle
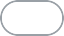
 ﻣﺗزوجMarried
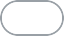
 ﻣطﻠق Divorced
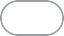
 أرﻣلWidowed
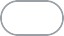


*اﻟﺣﺎﻟﺔ اﻟوظﯾﻔﯾﺔstatus. 4. Employment

*Mark only one oval. اختار اجابة واحدة فقط*

اﻟﻘطﺎع اﻟﺧﺎصsector Private
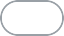
 ﻗطﺎعﺣﻛوﻣﻲsector government
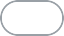


ﻏﯾر ﻋﺎﻣﻠﯾنemployed non
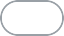


*ھلﺗدﺧن؟ ﺳﯾﺟﺎرة أوﻓﯾب أواﻷرﺟﯾﻠﺔpipewater? or vape or cigarette smoke you 5. Do

*Mark only one oval. اختار اجابة واحدة فقط*


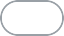
Yes نعم
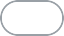
No لا

6. Date of onset of symptoms (dd/mm/yyyy), when did the symptoms start?

تاريخ ظهور الاعراض (يوم-شهر-سنة)

*Example:7January 2019*

*مثال: 7-1-2019*

*ھل شفيت تماما؟7. Have you made full recover?

*Mark only one oval. اختار اجابة واحدة فقط*


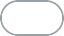
Yes نعم
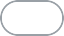
No لا

*ھل ﻣﺎزﻟت ﺗﻌﺎﻧﻰ ﻣن اﻷﻋراض؟8. Are you still troubled with symptoms

*Mark only one oval. اختار اجابة واحدة فقط*


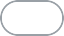
Yes نعم


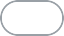
No لا

*ھل اﻷﻋراض ﺗﺗﻘﻠب ﻓﻲ ﺷدﺗﮭﺎ؟9. Do your symptoms fluctuate in severity.

*Mark only one oval. اختار اجابة واحدة فقط*


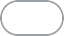
Yes نعم
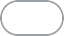
Noلا

*ھل ﺗﺗﺣﺳن أﻋراﺿك ﺑﺎﻷدوﯾﺔ؟10. Do your symptoms improve with medication

*Mark only one oval. اختار اجابة واحدة فقط*


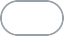
Yes نعم
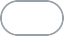
Noلا

*ھل ﺗﺗﺣﺳن أﻋراﺿك ﺑﺎﻟراﺣﺔ؟11. Do your symptoms improve with rest

*Mark only one oval. اختار اجابة واحدة فقط*


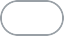
Yes نعم
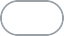
No لا

12. Have you ever had to stay in hospital because of COVID-19 symptoms? *

ھل ﺳﺑق ﻟك أن ﺑﻘﯾت ﻓﻲاﻟﻣﺳﺗﺷﻔﻰ ﺑﺳﺑب أﻋراض كورونا؟

*Mark only one oval. اختار اجابة واحدة فقط*


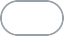
Yes نعم
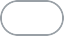
No لا

COVID-19 Symptoms 19 continued...

*ﻣﺎھﻲ ﻣدة اﻻﻗﺎﻣﺔ ﺑﺎﻟﻣﺳﺗﺷﻔﻰ؟13. How long did you stay in the hospital.

*Mark only one oval. اختار اجابة واحدة فقط*

أﻗل ﻣن أﺳﺑوعLess than a week
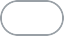


1-2أﺳﺑوع 1-2weeks
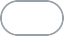


أﻛﺛرﻣن أﺳﺑوﻋﯾن More than 2 weeks
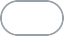


*ھل اﺣﺗﺟت إﻟﻰ أي ﻧوع ﻣن أﻧواع اﻟدﻋم14. Did you need any respiratory support?

اﻟﺗﻧﻔﺳﻲ؟

*Mark only one oval. اختار اجابة واحدة فقط*


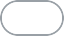
Yes نعم
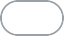
No لا

*ھل اﺣﺗﺟت ﻟﻠﺑﻘﺎء ﻓﻲوﺣدة اﻟﻌﻧﺎﯾﺔ15. Did you need to stay in the intensive care unit?

؟(ICU) اﻟﻣرﻛزة

*Mark only oneoval. اختار اجابة واحدة فقط*


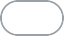
Yes نعم
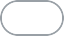
No لا

COVID-19 Symptoms 19 continued..

1. How long have you had COVID-19 symptoms overall? *

كم المدة التي عانيت فيها من اعراض الكورونا؟

*Mark only oneoval. اختار اجابة واحدة فقط*

أﻗل ﻣن أﺳﺑوﻋﯾنLess than 2 weeks
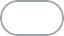


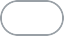
2-3 weeks 2-3 اسابيع


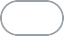
4-12 weeks 4-12 اسبوع

أﻛﺛرﻣن12 أﺳﺑوًﻋﺎMore than 12 weeks
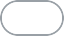


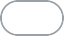
Noneلا شيء

1. How many times you were infected with Covid19? *

كم مرة اصبت بكورونا

*Mark only one oval. اختار اجابة واحدة فقط*


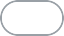
1


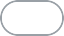
2


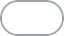
3


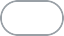
4


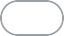
5

1. Which of the following symptoms were new or worse after you had COVID-19*

أي ﻣن اﻷﻋراض اﻟﺗﺎﻟﯾﺔ ﻛﺎن ﺟدﯾًدا أوأﺳوأ ﺑﻌد إﺻﺎﺑﺗك ﺑـﻛورونا وﻻ ﺗزال ﻣوﺟودة؟and are still present?

*Tick all that apply.اختار كل ما ينطبق*

Shortnessof breath

ضيق تنفس

Never

ﻣطﻠﻘﺎ

New

ﺟدﯾد

Still presentﻻ ﺗزال ﻣوﺟودة

ﺳﻌﺎﺎل Cough

Sore throat

التهاب الحلق

Palpitations

الخفقان

Joint and muscle ache

الام مفاصل و عضلات

Muscle stiffness

تصلب العضلات

Loss of smell

فقدان الشم

Sleep disturbance

اضطراب النوم

Memory problems

مشاكل في الذاكرة

Concentration problems

مشاكل في الذاكرة

Cognitive dysfunction

مشاكل في الادراك

ﺻداعHeadache

Chest pain

الم في الصدر

Vision disturbance

اضطرابات بصرية

Hormonal disturbances

اضطرابات هرمونية

طﻧﯾنTinnitus

Sweat odor

رائحة تعرق

Nausea/ vomiting

الغثيان او القىء

Rashes

الطفح اﻟﺟﻠدي

Tremors

الرعاش

نوبات الصرع Seizures

Lack of motivation

نقص الحافز

Mental health problems

مشاكل في اﻟﺻﺣﺔ اﻟﻌﻘﻠﯾﺔ

Decrease in appetite

ضعف في الشهية

1. Please answer “Yes” or “No” to the following as they apply to you:(medical*

اﻟرﺟﺎء اﻹﺟﺎﺑﺔ ﺑـ "ﻧﻌم" أو "ﻻ" ﻋﻠﻰ ﻣﺎ ﯾﻠﻲ ﻛﻣﺎ ﯾﻧطﺑق ﻋﻠﯾك:)اﻟﺗﺎرﯾﺦ اﻟطﺑﻲ(history).

*Mark only one ova lper row. اختار اجابة واحدة فقط لكل سطر*

Yes No

اﻟرﺑوAsthma

Arthritis

التهاب المفاصل

Migraine headache ﺻداع ﻧﺻﻔﻲ

Diabetes

السكري

اﻟﻟﺻرع Epilepsy

Bronchitis

التهاب الشعيبات الهوائية

Heart Disease

ﻣرضﻗﻠﺑﻲ

Stomach/intistinal ulcers

قرحة المعدة والامعاء

Stroke effects

آﺛﺎر ااﻟﻟﺳﻛﺗﺗﺔ الدماغية

Dementia

اﻟﺧرف

Food allergies

ﺣﺳﺎﺳﯾﺔ الطعام

اﻟﻟﺳﻣﻧﺔObesity

اﻟﻌﻼﺟﺎتوالادوية المتناولةMedication and treatment history

1. Have you used or currently using any type of vitamins and supplements? *

ھل اﺳﺗﺧدﻣت أوﺗﺳﺗﺧدم ﺣﺎﻟًﯾﺎ أي ﻧوع ﻣن اﻟﻔﯾﺗﺎﻣﯾﻧﺎت أو اﻟﻣﻛﻣﻼت اﻟﻐذاﺋﯾﺔ؟

*Tick all that apply.اختار كل ما ينطبق*

Vitamin A
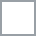
Vitamin C
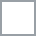
Vitamin E
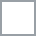
Vitamin K
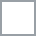
Vitamin D
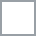
Vitamin B1
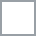
Vitamin B6
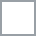
VitaminB12
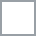


Calcium supplement
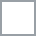
Zinc supplement
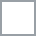


Mg supplement
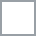
Mn supplement
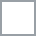
None

ﺗﺎرﯾﺦاﻟﺗطﻌﯾمHistory Vaccination

*ھل ﺗﻠﻘﯾت اﻟﺟرﻋﺔ اﻷوﻟﻰ ﻣن ﻟﻘﺎح كورونا21. Have you had the first dose of COVID vaccine?

.

*Mark only one oval. اختار اجابة واحدة فقط*


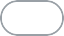
Yes نعم
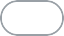
Noلا

1. What is the name of the first dose ‘s vaccine you received? *

ما اسم مطعوم الجرعة الاولى الذي تلقيته؟

*Mark only one oval. اختار اجابة واحدة فقط*

أﻛﺳﻔوردأﺳﺗرازﯾﻧﯾﻛﺎAstraZenecaOxford
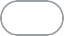


ﻓﺎﯾزرﺑﯾوانﺗكBioNTechPfizer
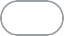


Sputnik vaccineالروسي


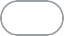


- Sinopharm vaccine اﻟﺻﯾﻧﻲ


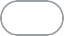


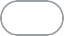
None

1. Date of first dose vaccine (dd/mm/yyyy). ﻟﻘﺎح ﺟرﻋﺔ أول ﺗﺎرﯾﺦ

*Example:7 January 2019*

*مثال: 7-1-2019*

1. Have you had the second dose of the COVID-19 vaccine? *

هل تلقيت الجرعة الثانية من لقاح كورونا؟

*Mark only one oval. اختار اجابة واحدة فقط*


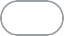
Yes نعم
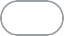
Noلا

1. What is the name of the second dose‘s vaccine you received? *

ما اسم مطعوم الجرعة اﻟﺛﺎﻧﯾﺔ الذي تلقيته؟

*Mark only one oval. اختار اجابة واحدة فقط*

أﻛﺳﻔوردأﺳﺗرازﯾﻧﯾﻛﺎAstraZenecaOxford
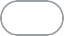


ﻓﺎﯾزرﺑﯾوانﺗكBioNTechPfizer
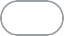


- Sputnik vaccine الروسي


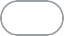


- sinopharmvaccineاﻟﺻﯾﻧﻲ


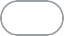


- None


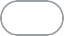


1. Date of second dose vaccine (dd/mm/yyyy). ﻟﻘﺎح ﺟرﻋﺔ ثاني ﺗﺎرﯾﺦ

*Example:7 January 2019*

*مثال: 7-1-2019*

1. Have you had the third dose of the COVID-19 vaccine (booster shot)? *

هل تلقيت الجرعة الثالثة (المعززة) من لقاح كورونا؟

*Mark only one oval. اختار اجابة واحدة فقط*


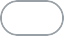
Yes نعم
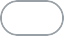
Noلا

Symptoms recorded after vaccinationاعراض بعد اللقاح

1. Did you experience any symptoms after the Covid-19 vaccine? *

هل واجهت اي اعراض بعد المطعوم

*Mark only one oval. اختار اجابة واحدة فقط*


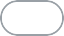
Yes نعم
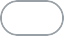
Noلا

*ھلﻋﺎﻧﯾت ﻣن اﻟﺗﻌب واﻹرھﺎق؟28. Have experience fatigue or tiredness

*Mark only one oval. اختار اجابة واحدة فقط*


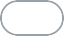
Yes نعم
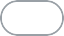
No لا

*ھلﻋﺎﻧﯾت ﻣن اﻟﺣﻣﻰأواﻟﺻداع؟headache or fever experienced you Have 29.

*Mark only one oval. اختار اجابة واحدة فقط*


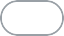
Yes نعم
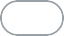
Noلا

30. Have you experienced pain or swelling at the injection site? *

هل عانيت من الم او ﺗورم ﻓﻲ ﻣوﻗﻊ اﻟﺣﻘن؟

*Mark only oneoval. اختار اجابة واحدة فقط*


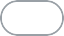
Yes نعم
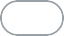
Noلا

*ھل ﺷﻌرت ﺑﺄﻟم ﻓﻲاﻟﻣﻔﺎﺻل؟pain joint felt you 31. Have

*Mark only one oval.*


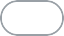
Yes نعم
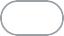
Noلا

*ھلﻋﺎﻧﯾت ﻣن أﻟم ﻋﺿﻠﻲ pain? ((muscle myalgia experienced you Have 32.

*Mark only oneoval. اختار اجابة واحدة فقط*


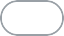
Yes نعم
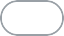
Noلا

*ھلﻋﺎﻧﯾت ﻣن اﻟﻐﺛﯾﺎن؟33. Have you experienced nausea

*Mark only one oval. اختار اجابة واحدة فقط*


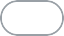
Yes نعم
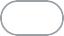
Noلا

*ھلﻻﺣظت أي ﻛدﻣﺎت ﻋﻠﻰ ﺟﺳﻣك؟body your on bruises any noticed you Have 34.

*Mark only one oval. اختار اجابة واحدة فقط*


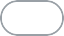
Yes نعم
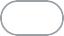
Noلا

*ھل ﺷﻌرت ﺑﺎﻟدوار؟dizzy felt you Have 35.

*Mark only one oval. اختار اجابة واحدة فقط*


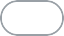
Yes نعم
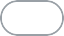
Noلا

*ھل ﻻﺣظت ﺗﺳﺎرع ﺿرﺑﺎت اﻟﻘﻠب أوﻋدمheartbeats? irregular or faster noticed you Have 36.

اﻧﺗظﺎﻣﮭﺎ؟

*Mark only one oval. اختار اجابة واحدة فقط*


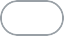
Yes نعم
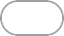
Noلا

37. Have you experienced an increase or decrease in blood pressure? *

هل عانيت من ارﺗﻔﺎع أو اﻧﺧﻔﺎض ﻓﻲﺿﻐط اﻟدم؟

*Mark only one oval. اختار اجابة واحدة فقط*


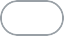
Yes نعم
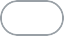
Noلا

[
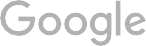
Forms](https://www.google.com/forms/about/?utm_source=product&utm_medium=forms_logo&utm_campaign=forms)
